# Validation of the professional good care scale in nursing homes (GCS-NH)

**DOI:** 10.1186/s12877-021-02199-6

**Published:** 2021-04-15

**Authors:** Gema Pérez-Rojo, Javier López, Cristina Noriega, José Angel Martínez-Huertas, Cristina Velasco

**Affiliations:** 1 Department of Psychology and Pedagogy, School of Medicine, Universidad San Pablo-CEU, CEU Universities, Campus de Montepríncipe, 28925 Alcorcón, Madrid, Spain; 2 Department of Cognitive Psychology at the Universidad Autónoma de Madrid, Madrid, Spain

**Keywords:** Institutional, Elder abuse, Protective factors

## Abstract

**Background:**

There is extensive concern about older people’s care in institutions, especially recently in the past years. One of the reasons is linked to the cases of elder abuse, not only shown by academic and scientific sources, but also by social and mass media and their impact on public perception of the institutional setting. What is more, current COVID-19 pandemic consequences on older people have provoked alarm and worry especially about what is happening in institutions.

**Methods:**

The sample for this study consists of 286 staff working in nursing homes in Spain. This study aimed to assess the psychometric properties of the Professional Good Care Scale in Nursing Homes (GCS-NH).

**Results:**

Results of parallel analyses and exploratory factor analyses (EFAs) showed a four-factor model for the 32-item scale: *humanization* (9 items), *non-infantilization* (10 items), *respect* (7 items) and *empowerment* (6 items). Then, psychometric properties were tested analysing internal consistency (reliability) and convergent, divergent and criterion validity. High internal consistency (reliability) and different validity evidence were obtained for the total scores of the GCS-NH and its subscales. GCS-NH scores were also capable of detecting risk of probable institutional elder abuse.

**Conclusions:**

Results show that this scale is an appropriate, valid, and reliable multidimensional instrument to evaluate good care in older institutionalized people by staff. Good care is an outcome of a complex construct in which a wide range of factors converge (staff, older people, and environmental characteristics). The GCS-NH has potential to be used as a multidimensional tool to assess good care.

**Supplementary Information:**

The online version contains supplementary material available at 10.1186/s12877-021-02199-6.

## Background

There is no doubt that aging is a worldwide reality. Demographic trends indicate that the population group of people aged over 65 will continue to rise, and especially so in people over 85 years of age. This demographic change, along with other economic and social changes, may have several consequences for older people. Despite the fact that there is not a causal relationship between age and dependency and/or illness, the older in age, the more probability there is of health problems, as well as a greater likelihood of needing assistance in daily living and long-term care, decreased levels of social support and more difficulties in accessing health care. These characteristics increase older people’s vulnerability, making them have higher risk of abuse and/or neglect [1].

Caregivers experience high levels of burden, anxiety, and depression [2]. The institutionalisation of a relative is not an improvised decision; on the contrary, it is often the result of long reflection and consultation [3]. There is extensive concern about older people’s care in institutions, especially over the last few years. One of the reasons is linked to the cases of elder abuse shown not only by academic and scientific sources, but also by social and mass media and their impact on public perception of the institutional setting [4]. What is more, current COVID-19 pandemic consequences on older people have provoked alarm and worry about what is happening in these institutions [5].

Most institutionalized users have cognitive and/or functional impairment and might show disruptive behaviours like wandering, aggressive behaviour or agitation [6]. Taking care of people with these characteristics is a demanding and backbreaking task that can be overwhelming for staff. Feeling overloaded can have negative consequences on both residents and professionals, especially if professionals do not have enough training and are not well-prepared [1]. This, in turn, may affect the quality of care and treat given to residents [7]. In fact, the quality of care and treatment received is associated with staff characteristics: socio-demographic information (age, subjective health, educational level), working conditions and organizational variables: average time working, satisfaction and motivation with their work, time pressure, overload and available resources, person-centred care and person-directed care, and older characteristics like frequency of behaviour problems [8, 9].

Studies about the quality of care in institutions by staff are scarce [10] and they are mostly focused on elder abuse and explicit categories such as physical, psychological, financial, neglect and sexual [11]. To know this phenomenon in-depth, it is necessary to have valid and reliable instruments. However, this aspect is underdeveloped in institutions [12]. There are a few instruments that assess elder abuse by interviewing professionals who work at institutions. For example, Pillemer and Moore [13] used an instrument based on the Conflict Tactics Scale to assess physical and psychological abuse, and Hsieh et al. [14] developed the Caregiver Psychological Elder Abuse Behaviour Scale (CPEAB) which is focused on identifying elder abuse informed by nursing home staff. However, most research analysing elder abuse in institutions has used instruments without reporting any psychometric properties [12].

These instruments consider the public and professional perspective, but not older adults’ main concerns. For older people, disrespect is the most painful kind of elder abuse and the cause of the rest of the types of harm [15]. Disrespect is linked, on one hand, to violation of human rights such as dignity, privacy, or autonomy and, on the other hand, to infantilization [16].

According to this idea, Kayser-Jones [17] purposed a theoretical frame of reference about quality of care in long-term care facilites defining four types of institutional abuse: depersonalization (delivering care services to the residents according to basic requirements that overlooks their peculiarities), dehumanization (disregarding their privacy, their dignity and autonomy to make decisions), infantilization (treating them like children), and victimization (threats, intimidation, and physical or verbal injury or harm). This framework is consistent with strategy and action plan to prevent elder abuse developed by WHO [18]. It focuses on the establishment of person-centred perspective to promote residents´ quality care in long-term institutions and protect their rights.

This approach follows the same direction as the significant worldwide change experienced by the long-term care model, from task-oriented and professional-driven (institution-centred perspective) to a person-centred care perspective that focuses on individual preferences and necessities [19]. This reflects a movement from the biomedical approach, based on clinical quality care, to the biopsychological one, based on people’s quality of life and quality of caring [20]. The latter is based on a humanistic perspective widely developed in gerontology settings in the past years [21]. This perspective changes the mindset when dealing with institutional elder abuse, because it is a sensitive issue, as it considers nursing homes’ performance more extensively, avoiding focusing on elder abuse exclusively, with the stigmatization that this entails [4]. On the contrary, it focuses on the promotion of good daily care practices, such as respect, humanization, and ethical values [1].

However, there are few instruments with suitable psychometric properties to measure this construct and the available tools do not take into account the most harmful categories for older people; the implicit ones such as disrespect. For this reason, the present study sought to fill the existing gap in the related research by providing a new self-reported valid and reliable instrument based on the Kayser-Jones´ conceptual framework [17]. Previously, the content validity of the scale was analysed to develop a new instrument of international interest, for both clinical and research fields [22]. But it is crucial for the instrument to show suitable empirical evidence of validity and reliability. For this reason, this study carries out the validation process exploring the psychometric properties of the GCS-NH and its factor structure in Spanish staff working in institutions.

## Methods

### Participants

The sample for this study consists of 313 nursing home staff members from 12 facilities in Spain. Data was collected from private (*N* = 67; 21.40%), for-profit (*n* = 178; 56.90%), and public (*N* = 62; 19.80%) nursing homes. The eligibility criteria for the study included people who had been working in nursing homes for a minimum of three months and were 18 years old or more. Of the 313 nursing home professionals contacted, 27 did not meet the criteria of working on the front line (staff directly involved in care). As a result, the sample was reduced to 286 formal caregivers. 86.36% of the final sample were women (*N* = 247) and the average age was 39.21 years old (*SD* = 12.15) ranging from 19 to 70 years of age. The average time working in the nursing home was 71.01 months (*SD* = 17.81), 96.50% reported medium or high satisfaction with their work (*N* = 276), and 89.16% reported good or very good physical health (*N* = 255).

### Instruments

*Sociodemographic* and professional variables: age, sex, educational level, subjective health, average time working, satisfaction and motivation with their work, time pressure, overload and available resources.

*Professional Good Care Scale in Nursing Homes (GCS-NH)*. The content of this scale is in line with the perspective of centred care that enhances the need of providing individualized care, in which older people’s specific needs are met without carrying out relationships of power. As it was stated in the introductory section, the scale was developed to measure four constructs and the content validity of the items was evaluated by different experts in a previous study [22]. Staff members report the frequency level a list of behaviors related to work with older adult in institution have been carried out by them in the last month on a scale from 0 “A lot, if it has been carried out more than 10 times in the last month” to 4 “Never, if it has not been carried out in the last month. In the *Results* section, the empirical factor structure and different evidence of reliability and validity of the scale are presented.

*The Revised Memory and Behaviour Problems Checklist- Nursing Homes* (RMBPC-NH [23];). The Spanish version was used [24]. This 42-item instrument was used to measure the frequency of residents´ behaviour problems (cognitive, emotional, functional and other problems) and its relationship with staff’s burden and other residents´ well-being. The scale has high reliability (Cronbach’s α = .96; McDonald’s ω = .96).

*The Staff Assessment Person-Directed Care* (PDC). The PDC [25] is a self-assessment tool consisting of 50 items with five-point Likert-type, which measures the level of person-centred care provided by front-line care workers. The tool contains two main factors: Person-Directed Care and Environmental Support for Person-Directed Care. The first factor comprises five subscales: autonomy, personhood, knowing the person, comfort care and support relations. The second one includes work with residents, personal environment for residents and management/structure. The Spanish version [26] showed good reliability in our sample (Cronbach’s α = .95; McDonald’s ω = .96).

*The Person-centred Care Assessment Tool* (P-CAT) [27]. The Spanish version of P-CAT was used [28], which includes 13 items with five-point Likert-type that measure the level of person-centred care delivered by front-line care workers. This tool evaluates three factors: personalizing care, organizational support and environmental accessibility. The Spanish version showed good reliability in our sample (Cronbach’s α = .80; McDonald’s ω = .81).

### Procedure

We contacted with managers of licensed nursing homes in Spain and gave them verbal and written information about the nature and the goals of the research, the procedure and the methodology to be undergone, and invited them to take part in the study. Twelve facilities agreed to participate. A set of inclusion criteria was used: eligible participants were aged 18 or older, had been working at the centre for at least 3 months, and were providing direct care for users. Staff that met the eligibility criteria was asked if they were interested in participating in the study.

Participation was voluntary, and no reward was offered for the collaboration. Informed consent was obtained from all respondents, and confidentiality was explicitly guaranteed. They were also informed that they had the right to withdraw from the study at any time. The study was approved by the Spanish Ministry of Economy and Competitiveness and the University CEU San Pablo Ethics Committee. All methods were performed in accordance with the relevant guidelines and regulations such as the Declaration of Helsinki.

Participants were asked to fill a self-administered questionnaire which included staff characteristics (sex, age, subjective health and educational level), working conditions and organizational variables (average time working, satisfaction and motivation with their work, time pressure, overload and available resources), person-centred care and person-directed care, and residents´ characteristics (frequency of behaviour problems). Participants filled the assessment protocol on their own, but they could ask a trained psychologist from the research team if they had any questions.

### Data analysis

All the statistical analyses were performed using R software [29]. First, seven items were removed from the initial sample of items because they gathered more than 90% of responses into the same item category (i.e., very small variability was observed in those items as almost all the subjects answered the ‘*Strongly agree*’ category). Secondly, different exploratory factor analyses (EFAs) were conducted with *Unweighted Least Squares* (ULS) and *oblimin* rotation using R’s *psych* package [30] due to the fact that no previous factorial structure has been empirically tested for this scale. An iterative process, combining EFAs and evaluating the underlying number of factors with parallel analyses (scree plots), was followed to remove those items that presented low factor loadings (i.e., factor loadings lower than .30). Then, the initial sample of 49 items was reduced to 32 items. Model fit of EFAs was evaluated with standard criteria (e.g., RMSEA equal to or below .06, SMSR equal to or below .08 [31–33];. As the *oblimin* rotation showed that the correlations of the estimated factors were small, a *varimax* rotation was applied (no relevant changes were observed in the estimated factor loadings). In third place, descriptive and reliability (Cronbach’s α, and McDonald’s ω) results were computed for the scale and its subscales. In fourth place, Pearson correlation coefficients with relevant variables were used to examine the convergent and discriminant validity of the scale and its subscales. In fifth place, the capacity of detecting institutional elder abuse was evaluated. To do so, values lower than the 25-percentile of the Spanish adaptation of the P-CAT scale were classified as probable or suspicion of institutional elder abuse [26]. In this way, different logistic regression models (logit link) and receiver operating characteristic curves were computed to analyse their capacity to evaluate the presence of institutional elder abuse. The logistic regression model was estimated with R’s base functions, and receiver operating characteristic curve was estimated using R’s *precrec* package [34].

## Results

### Descriptive analyses and factor structure of items

Parallel analyses (scree plots) show that there are four underlying factors as was previously determined by expert judges when the scale was developed [22]. This result was obtained in all the steps of the iterative process used to remove items that did not have a factor loading equal to or above .30. The final scale was composed of 32 items. The four-factor model showed an adequate fit to the data of the final set of items: χ^2^(374) = 634.70, *p* < .001, RMSEA [90%IC] = .049 [.043–.056], RMSR = .05, BIC = − 1480.64. The standardized factor loading structure of this model can be seen in Table 1. Also, descriptive analyses (mean and standard deviation) were conducted for the items. As can be observed, all the item means, and their factor loadings were appropriate, and no relevant cross-loadings were observed. A four-factor solution was obtained for the 32-item scale (eigenvalues ranged from 5.08 to 1.47) explaining 32% of the variance.

**Table 1:** Descriptive statistics (mean and standard deviation) and standardized factor loading structure of the Professional Good Care Scale in Nursing Homes (GCS-NH)

Item	Mean (*SD*)	Factor loadings				Item	Mean (*SD*)	Factor loadings			
		F1	F2	F3	F4			F1	F2	F3	F4
1	3.52 (.74)	**.53**	−.19	.07	−.03	17	2.45 (1.54)	.18	.01	−.05	**.47**
2	3.34 (.75)	**.36**	−.18	.01	.08	18	3.28 (.84)	**.55**	.14	.09	.23
3	1.89 (1.31)	−.07	**.47**	−.02	.12	19	2.36 (1.60)	−.13	**.61**	.05	−.01
4	3.26 (.85)	**.45**	.09	.02	.10	20	3.62 (.77)	**.48**	−.12	.22	.14
5	2.85 (1.18)	.26	.07	**.37**	.06	21	2.74 (1.40)	.00	**.48**	−.19	.10
6	2.62 (1.61)	−.01	**.43**	−.21	.14	22	3.53 (.73)	**.48**	−.03	.05	.19
7	2.35 (1.69)	**.34**	−.28	.14	.07	23	2.96 (1.35)	−.01	**.58**	.18	.07
8	3.21 (1.33)	.08	−.22	**.58**	.13	24	3.68 (.81)	**.43**	−.06	.09	−.04
9	2.05 (1.38)	.12	**.38**	.07	−.14	25	3.77 (.74)	−.09	**.33**	.24	−.05
10	3.18 (1.15)	.29	.03	**.54**	.11	26	2.52 (1.70)	.12	.19	.04	**.60**
11	3.40 (1.12)	.08	−.12	**.55**	.17	27	2.28 (1.44)	.20	−.05	.22	**.52**
12	3.65 (.94)	−.21	.29	**.51**	−.05	28	2.76 (1.56)	−.03	**.51**	−.13	−.03
13	3.45 (.82)	**.64**	−.01	.23	.09	29	2.46 (1.45)	.03	.16	.29	**.57**
14	3.45 (.89)	.34	.07	**.61**	.07	30	1.29 (1.24)	−.04	**.32**	.04	−.24
15	2.83 (1.47)	−.10	**.49**	.14	−.05	31	2.49 (1.52)	−.01	−.10	.04	**.59**
16	3.39 (1.08)	.24	.04	**.60**	.07	32	3.09 (1.34)	.14	−.01	.11	**.56**

*N* = 286. *SD* = Standard deviation. F1 = Humanization. F2 = Non infantilization. F3 = Respect. F4 = Empowerment. ULS estimator and *varimax* rotation were applied to estimate factor loadings. Bold numbers show the highest factor loading for each item. Scores ranged from 0 (Strongly disagree) to 4 (Strongly agree). Items in italics are reverse-coded

The first factor includes nine items related to bonding, connection, tenderness, closeness (e.g. “Looking after users´ requests as soon as possible”). All items are positively scored. This factor was labelled *humanization*. The second factor contains ten items and mainly reflects the consideration of older people as adults; avoiding the older adult being treated like children and any overprotection by the staff members (e.g. “*Punishing users as if they were children*”). In this case, all items are inversely scored. This factor was labelled *Non-infantilization*. The third factor is made up of seven items reflecting respect and avoiding stigmatization by staff (e.g. “Maintaining discretion over users´ personal issues.”). This factor was labelled *Respect,* and all its items are positively scored except item 12, which is negatively scored. The fourth and final factor includes six items about residents´ decision-making and choices and having control over their lives (e.g. “Offering the user the choice of napping during the day”). This factor was labelled *empowerment*, and all its items are positively scored. The total score on the GCS-NH consists of the sum of the scores of all the subscales. A higher score reflects better care.

### Descriptive analysis and reliability

Table 2 presents the descriptive analysis and the reliability of the GCS-NH and its subscales. As can be observed, all the variables obtained desirable reliability properties, especially considering the small number of items of some subscales. All the variables obtained relatively large mean scores, being the *Non-infantilization* and the *Empowerment* subscales the ones presenting a medium mean score (2.51 and 2.57 out of 4, respectively). This means that, in general, the GCS-NH scores tend to present a negatively skewed (left-skewed) distribution.

**Table 2:** Descriptive statistics and reliability of the GCS-NH and its subscales

Variable	Descriptive analysis					Reliability	
	Items	M	SD	Mdn	Range	Cronbach’s α	McDonald’s ω
Total	32	2.93	.42	2.97	1.41–3.87	.77	.83
Humanization	9	3.34	.52	3.44	.89–4.00	.75	.75
Non-infantilization	10	2.51	.74	2.56	.67–3.90	.72	.72
Respect	7	3.30	.71	3.43	.00–4.00	.76	.77
Empowerment	6	2.57	1.01	2.83	.00–4.00	.74	.75

*N* = 286. Items = number of items; M = mean; SD = standard deviation; Mdn = median. Item scores ranged from 0 (Strongly disagree) to 4 (Strongly agree)

### Convergent and discriminant validity

Table 3 presents Pearson correlation coefficients as evidence of the convergent and discriminant validity of the scores of the GCS-NH and its subscales. As can be observed, the scores of the GCS-NH presented statistically significant negative relations with age, subjective health, the frequency of the older adult’s behaviour problems, average time working, time pressure, overload, available resources. On the contrary, they show significant positive relations with satisfaction with their work, motivation, educational level, and the level of person-centred and person-directed care provided. In general, non-infantilization and empowerment subscales presented higher correlation coefficients than humanization and respect.

**Table 3:** Pearson correlation coefficients of the Professional Good Practices Scale in Nursing Homes (GPS-NH) and its subscales with different variables

Variable	Total	Humanization	Non-infantilization	Respect	Empowerment
Age	−.12^*^	−.03	−.09	−.12^*^	−.04
Subjective health	−.17^**^	.07	−.25^**^	−.04	−.09
Satisfaction with their work	.20^**^	.23^**^	.10	.01	.13^*^
Average time working	−.13^*^	.08	−.20^**^	.00	−.10
Time pressure	−.14^*^	.05	−.32^**^	.13^*^	−.07
Overload	−.20^**^	.00	−.33^**^	.11	−.13^*^
Insufficient resources	−.21^**^	.07	−.37^**^	.10	−.15^*^
Motivation	.23^**^	.21^**^	.07	.04	.22^**^
Educational level	.21^**^	.09	.16^**^	.05	.15^*^
P-CAT	.38^**^	.12^*^	.32^**^	.09	.27^**^
PDC	.39^**^	.11	.28^**^	.11	.33^**^
Behavioral problem frequency	−.13^*^	−.05	−.20^**^	.02	−.03

*N* = 286. ** = *p* < .01. * = *p* < .05

### Detecting risk of probable institutional elder abuse

One of the most relevant validity evidences of the GCS-NH is the capacity to detect probable institutional elder abuse. A logistic regression model (logit link) was conducted to detect institutional elder abuse using the GPS-NH score. The presence of risk of institutional abuse was computed according to the classification of the P-CAT (in this study: 0 = abuse; 1 = no abuse). The estimated intercept of the model was − 3.84 (*SE* = 1.14) with a z-value of − 3.37 (*p* < .001), and the estimation for the GPS-NH score was 1.91 (*SE* = .41) with a z-value of 4.65 (*p* < .001). Furthermore, the GPS-NH score obtained an area under the curve from receiver operating characteristic curves equal to .73 when classifying risk of probable institutional elder abuse. The results of both the logistic regression model and the receiver operating characteristic curve mean that the GPS-NH is capable of detecting institutional elder abuse (the higher the GPS-NH score is, the lower the probability of institutional elder abuse).

## Discussion

There is a lack of suitable instruments with good psychometric characteristics to measure older’s good care received by staff working in institutions. For this reason, we set out to validate and analyse the psychometric characteristics of a new tool for measuring good care of institutionalized older adult by staff, namely GCS-NH. Our results provide satisfactory evidence for the reliability and validity of the GCS-NH. Consistent with the standards for psychological testing, our tool showed an appropriate content validity, internal structure, and convergent validity [35].

We used parallel analyses and EFAs to analyse the underlying factor structure of the scale. Results supported a multidimensional structure composed of four factors. The dimensions were *humanization*, *non-infantilization*, *respect* and *empowerment*. Thus, it seems that good care or good treatment is a multidimensional concept. Although the final factors are not exactly the same as the ones Kayser-Jones [17] proposed, they are in the same line as her idea of independence, freedom and making choices such as quality care key elements [36]. In the last few years, one of the main aims regarding older adult has been the promotion of activating their participation through empowerment [37].

Furthermore, results provided good support for the convergent and discriminant validity with other significant variables. Concerning convergent validity, we found a positive relationship between the GCS-NH (total score and some of subscales scores) and P-CAT and PDC. On one hand, P-CAT is related to all the subscales except *Respect* subscale and, on the other hand, PDC is related to *Non-infantilization* and *Empowerment*. These results suggest that GCS-NH good care factors are not covered completely by the content of these scales. Person-centred care and good care are wide constructs with relevant joint points. However, they are not the same [38].

In this study, evidence of validity based on the relationship with other significant sociodemographic variables like age, perceived health and educational level has also been proved. Concerning staff members ages, older staff reported less good care which is in line with the study carried out by Radermacher [39]. However, contradictory results have been found in other studies. Some of them found that younger staff provided worse care [9], while others reported no significant relationship between age staff and good care [8].

Regarding subjective health, care workers with better perceived health showed better care. Caring for residents with cognitive and functional impairment is a demanding task that requires staff in the best of conditions. For this reason, staff members with poorer perceived health might have more difficulties handling complicated situations in nursing homes [9].

In a similar way, there was a negative significant relationship between the frequency of problem behaviours and good care. It might reflect the staff’s difficulties in dealing with dementia consequences, like disruptive behaviours [8]. Another relevant result was that staff members who were more satisfied and motivated with their work were able to provide the quality of care necessary for older adults as was mentioned in other studies [40, 41]. Also, the relationship between organizational variables and the care that older adults receive in an institution, (such as time pressure, overload, insufficient resources and average time working), has been supported by literature [9, 42]. Our study showed that poor working conditions are highlighted as possible variables related with worse care provided by staff. The scientific literature has shown contradictory results with the average time working variable. Some research supports our results; these show that longer time working is associated with worse care [43, 44]. However, other studies found that the relation between these variables is positive [45].

PDC and P-CAT scales are generally used internationally for the evaluation of care in nursing homes focusing on the person-centred care approach. Although P-CAT [28], PCD and GCS-NH scales do not measure exactly the same construct, they are related. While person-centred care is a more holistic construct, good care is more specific and less focused on environmental conditions. It is also worth noting that the GCS-NH scale includes, for the first time, specific items to analyse infantilization.

P-CAT was used to evaluate evidence of criterion validity to detect institutional elder abuse using the GPS-NH score. To this date, there is no gold standard or external criteria against which to check the scores provided by the GCS-NH. Although it is necessary to overcome this limitation, the results using P-CAT are good and may help in future studies finding a high external criterion. The European Commission’s PACTE Program (p. 45) [46] pointed out that “Elder abuse/mistreatment in residential setting means any action or negligence on the part of staff, relatives or relevant other which constitutes inappropriate treatment or which violates the rights of residents and to which the resident objects or could reasonably be expected to object”. Considering this definition, the GPS-NH scale is capable of detecting institutional elder abuse (the lower the GPS-NH score is, the lower the probability of institutional elder abuse).

The main contribution of the present study is the development of a reliable instrument that assesses good care based on the older adults’ main concerns about the most painful way of elder abuse [15, 16]. We consider older adult should be the leading role in their own life. Because of the current pandemic situation, many institutions and professionals talk about the necessity of a change in the long-term care model [5]. It is important to discuss with older adults from different settings and circumstances to know what they need and what they want. The main strength of this tool is that items are focused on aspects that can be meaningful for users; especially for older adult with high support needs and silent voices according to Bowers et al. [47] and this follows the same line as Blood’s results [48]. Older adult want housing alternatives and more control over their own lives and the quality of care they receive [4]. Older adult having control over how they lead their fuller lives and the care they receive was highlighted as crucial in the current pandemic situation [49]. What is more, these results are consistent with the iceberg analogy in which the most explicit categories of elder abuse are generally known (like physical, psychological, economic, etc.) but not the most subtle ones like ageism, infantilization and disrespect. The GCS-NH subscales included these implicit elements, allowing to detect, for example, disrespect (the most painful kind of elder abuse according to older adult) [15]. The results also revealed the importance of including characteristics of the staff members, the institution, and the resident because they have an impact over the care received. Compared with other quality care scales, GCS-NH includes a non-infantilization dimension which is not included in other tools. This scale also reflects the multidimensionality of quality of care and does not focus only on one of them, for example, residents´ quality of life and the opinion of direct care staff. Moreover, GCS-NH assesses specific daily practices, allowing operationalise the concept and measuring the frequency and intensity of each behaviour [50, 51]. Finally, the scale emphasizes not only technical quality elements but also overall human quality factors.

Several limitations need to be acknowledged. First, this is a cross-sectional study that does not allow us to establish temporal precedence and causal relationships. Longitudinal studies are needed to ensure the stability of these results. Furthermore, like other similar studies, our study is based on a convenience and nonprobability sample, which may not be representative of the whole Spanish population of staff directly involved in care. This fact limits the generalizability of the results. It would also be desirable to have an objective criterion; a gold standard, to use to detect institutional elder abuse. Despite these limitations, to our knowledge, our scale is the first to assess good care through a brief (including only 32 items across 4 dimensions), easy-to-use and parsimonious way focusing on good care modifiable factors from a new perspective.

## Conclusion

To conclude, the GCS-NH presents acceptable psychometric properties and may contribute to the development of sensitive and conceptually valid outcome measures for psychological intervention research [52]. For this reason, GCS-NH is an appropriate instrument to evaluate older’s adults care in institutions from a different perspective, that is, a protective-factor approach instead of a traditional risk-factor approach based on the older’ strengths and not on their weaknesses. The key aspects of good care or good treatment, from the older’s viewpoint, are covered, because they focus on protecting them from disrespect (violation of human rights such as dignity, privacy or autonomy and infantilization) [16]. The main practical implications of our findings are the following: addressing good care in interventions and helping staff members acknowledge and manage them and develop strategies to improve nursing home care from older’s perspective.

## Supplementary Information

**Additional file 1.**

## Data Availability

The datasets generated and/or analysed during the current study are available from the corresponding author on reasonable request.
