# Supplementary material for: Validation of the professional good care scale in nursing homes (GCS-NH)
Source: BMC Geriatr. 2021 Apr 15;21:251. doi: 10.1186/s12877-021-02199-6 (PMC8047553; doi:10.1186/s12877-021-02199-6)
Supplement: Supplementary file 1 — Additional file 1. [file 12877_2021_2199_MOESM1_ESM.docx]

Appendix: **Professional Good Care Scale in Nursing Homes (GCS-NH)**

Below is a list of some behaviours related with care to older people institutionalized. Please, tell us how often, in the last month, these behaviours have happened using the following options: 0 = never; 1 = once; 2 = sometimes (between 2 to five times); 3 = several times (between 6 to 10 times); 4 = Many (more than 10 times).

| 1. Atender a los usuarios de modo personalizado. [Assisting users *(older people)* in a personalized way.] |
| --- |
| 2. Atender a la mayor brevedad las peticiones de los usuarios. [Looking after users´ requests as soon as possible.] |
| 3. *Proporcionar cuidados a los usuarios con prisa.* [*Providing care to users in a hurry.*] |
| 4. Fomentar el papel activo de los usuarios en el centro. [Encouraging users to develop an active role in the centre.] |
| 5. Preguntar a los usuarios cómo quieren ser llamados antes de utilizar diminutivos (Carmencita, Juanito, etc.). [Asking users how they want to be called before using diminutives (Johnny, Maggie, etc.)] |
| 6. *Ocultar información relevante a los usuarios. [Hiding users’ relevant information.*] |
| 7. Hablar sin gritar a los usuarios. [Speaking without yelling at users.] |
| 8. Procurar que los usuarios vayan vestidos con su ropa (no con la de otros). [Ensuring that users are dressed in their clothes (not with others´ ones] |
| 9. *Llevar de la mano a los usuarios, aunque no lo necesiten. [Taking users by the hand, even if they do not need it.]* |
| 10. Procurar un plan individualizado de cuidados para cada usuario. [Procuring an individualized care plan for each user.] |
| 11. Respetar la intimidad de los usuarios durante el aseo, realizándose con la puerta cerrada. [Respecting users´ privacy during their personal cleanliness, taking place with the door closed.] |
| 12. *Castigar a los usuarios en caso de que no se comporten como se espera. [Punishing users if they do not behave as expected]* |
| 13. Explicar a los usuarios por qué se hacen las cosas. [Explaining users why things are done.] |
| 14. Apoyar el desarrollo de actividades voluntarias a las que los usuarios pueden decidir libremente si asistir o no. [Supporting the development of voluntary activities to which users can freely decide whether to attend or not.] |
| 15. *Mezclar las pastillas con los alimentos sin informar a los usuarios. [Mixing the pills with the food without informing the users.]* |
| 16. Mantener discreción sobre cuestiones personales de los usuarios. [Maintaining discretion over users´ personal issues.] |
| 17. Facilitar a los usuarios la llave de su armario. [Providing users the key of their closet.] |
| 18. Tener en cuenta la toma de decisiones de los usuarios. [Taking into account users´ decision making.] |
| 19. *Realizar el aseo de manera mecánica. [Carrying out the personal cleaning mechanically.]* |
| 20. Trasladar al usuario cuidadosamente. [Moving users carefully] |
| 21. *Utilizar materiales infantiles en las distintas actividades. [Using children's materials in the different activities.]* |
| 22. Manejar adecuadamente la situación (con calma y respeto), aunque el usuario trate inadecuadamente al profesional. [Managing the situation properly (calmly and respectfully), even if the user treats the professional improperly.] |
| 23. *Forzar a comer a los usuarios si su vida no está en peligro. [Forcing users to eat when their life is not in danger.]*  24. Respetar las pertenencias del usuario. [Respecting users´ belongings.] |
| 25. *Utilizar castigos como si fueran niños. [Punishing users as if they were children.]* |
| 26. Permitir que los usuarios capaces para ello firman su documentación. [Allowing capable users to sign their documentation.] |
| 27. Dejar al usuario la elección de dormir siestas durante el día. [Offering the user the choice of napping during the day.] |
| 28. *Aunque no sea necesario, poner babero a todos los usuarios en el comedor. [Although it is not necessary, put all users a bib in the dining room*] |
| 29. Informar explícitamente a los usuarios de los cambios en el tratamiento. [Informing users explicitly of the changes in their treatment.] |
| 30. *Dirigirse a los usuarios con palabras como cielo, cariño, mi amor o similares.* [*Addressing users with words like heaven, love,* [*sweetheart*](http://www.spanishdict.com/traductor/sweetheart) *or similar.*] |
| 31. Organizar el armario del usuario contando con su permiso. [Organizing users´ closet with their permission.] |
| 32. Permitir personalizar la habitación para que los usuarios se sientan más cómodos. [Allowing to customize the room so that users feel more comfortable.] |
| \|  \| \| --- \| \|  \| \|  \| \|  \| \|  \| \|  \| |
